# NUP-98 Rearrangements Led to the Identification of Candidate Biomarkers for Primary Induction Failure in Pediatric Acute Myeloid Leukemia

**DOI:** 10.3390/ijms22094575

**Published:** 2021-04-27

**Authors:** Vincenza Barresi, Virginia Di Bella, Nellina Andriano, Anna Provvidenza Privitera, Paola Bonaccorso, Manuela La Rosa, Valeria Iachelli, Giorgia Spampinato, Giulio Pulvirenti, Chiara Scuderi, Daniele F. Condorelli, Luca Lo Nigro

**Affiliations:** 1 Department of Biomedical and Biotechnological Sciences, Section of Medical Biochemistry, University of Catania, 95123 Catania, Italy; vincenza.barresi@unict.it (V.B.); virginia.dibella@studium.unict.it (V.D.B.); anna.privitera@phd.unict.it (A.P.P.); giorgiaspampinato@unict.it (G.S.); scuderi331@gmail.com (C.S.); 2 Cytogenetic-Cytofluorimetric-Molecular Biology Lab, 95123 Catania, Italy; nellinaandriano@yahoo.it (N.A.); paolabonaccorso@libero.it (P.B.); larosamanuela@libero.it (M.L.R.); valeria.iachelli@gmail.com (V.I.); giulpulv@hotmail.it (G.P.); lonigro@policlinico.unict.it (L.L.N.); 3 Center of Pediatric Hematology-Oncology, Azienda Policlinico–San Marco, 95123 Catania, Italy

**Keywords:** acute myeloid leukemia, chemotherapy resistance, omics technologies, transcriptome profile, dysregulated genes, NUP98-rearrangements

## Abstract

Conventional chemotherapy for acute myeloid leukemia regimens generally encompass an intensive induction phase, in order to achieve a morphological remission in terms of bone marrow blasts (<5%). The majority of cases are classified as Primary Induction Response (PIR); unfortunately, 15% of children do not achieve remission and are defined Primary Induction Failure (PIF). This study aims to characterize the gene expression profile of PIF in children with Acute Myeloid Leukemia (AML), in order to detect molecular pathways dysfunctions and identify potential biomarkers. Given that NUP98-rearrangements are enriched in PIF-AML patients, we investigated the association of NUP98-driven genes in primary chemoresistance. Therefore, 85 expression arrays, deposited on GEO database, and 358 RNAseq AML samples, from TARGET program, were analyzed for “Differentially Expressed Genes” (DEGs) between NUP98+ and NUP98-, identifying 110 highly confident NUP98/PIF-associated DEGs. We confirmed, by qRT-PCR, the overexpression of nine DEGs, selected on the bases of the diagnostic accuracy, in a local cohort of PIF patients: *SPINK2*, *TMA7*, *SPCS2*, *CDCP1*, *CAPZA1*, *FGFR1OP2*, *MAN1A2*, *NT5C3A* and *SRP54*. In conclusion, the integrated analysis of NUP98 mutational analysis and transcriptome profiles allowed the identification of novel putative biomarkers for the prediction of PIF in AML.

## 1. Introduction

In the last decades, treatment of patients affected by acute myeloid leukemia (AML) has been improved, increasing the event-free survival (EFS) and overall survival (OS) rates [1,2] A better knowledge of pathogenetic and clinical features of AML produces a more accurate stratification guiding appropriate induction and post-induction therapies, which nowadays are supported by biological strategies. Despite these data, the percentage of patients that are resistant to primary induction therapy is around 10–12% in the pediatric population [3] and 30–40% in adults with AML (OHSU study) [4]. Primary Induction Failure (PIF) is defined as a failure to achieve complete response after two cycles of high-dose chemotherapy. Classical induction therapy consisted of the ICE scheme: Idarubicin, an anthracycline; Cytarabine, an antimetabolite and pyrimidine analogue; and Etoposide, a topoisomerase inhibitor. The criteria used to define induction failure are related to morphological assessment of therapy response. The outcome of these patients remains very poor [5] and some of them are directly addressed to allogenic hematopoietic stem cell transplantation (Allo-HSCT) as salvage strategy [6]. Currently, the molecular basis for the lack of response to conventional induction therapies in childhood AML is still unclear. Brown et al. found that point mutation of *SETBP1*, *ASXL1* and *RELN* were significantly associated with primary resistance [7] and the latter two were already linked with an increased risk of relapse [8]. However, these defined genetic mutations were present in a small fraction of patients [7].

One of the larger studies on the molecular landscape of pediatric AML, showed that the mutational profile of pediatric AML is markedly different from that of adult cases [9]. Moreover, somatic structural DNA changes of pediatric AML differ from adult cases, with a large number of gene fusions observed primarily or exclusively in pediatric cases, suggesting a special role for structural variants in younger subjects [9,10].

McNeer et al. reported that the most frequent mutations in pediatric AML, mainly as consequence of genomic rearrangements, involved *NUP98*, *WT1*, *RUNX1*, *MLLT10*, *SPECC1* and *KMT2C* [11]. On the contrary, pediatric cases seldom show some of the common mutations observed in adult cases, such as mutations of *DNMT3A*, *TET2* and *IDH1/2* [12].

In several studies chromosomal translocations involving the gene encoding the Nucleoporin 98 and 96 Precursor or Nuclear Pore Complex Protein Nup98-Nup96 (*NUP98*) predict a poor prognosis in AML [9,10,13,14,15]. NUP98 rearrangements (NUP98r) appear in about 5–15% cases of pediatric AML [14,15] and, combined with *FLT3* or *WT1* gene mutations, produce the worst outcome in AML [16]. In addition, it is known that NUP98-rearrangements are associated with a common Homeobox–A,-B (*HOX-A,-B*) gene expression signature that increases genome-instability [14] cooperating also with Meis1 [17].

NUP98 is a member of nuclear pore complex that manages the transport of macromolecules inside and outside the nucleus, can targets the chromatin by its N-terminal and can regulates transcription. NUP98 fusion products involve the N-terminal of NUP98 protein and C-terminal of relative partner. Some of the most frequent NUP98 fusion partners include HOX family members (*HOXA7* [t 7;11], *HOXA9* [t 7;11], *HOXA10* [t 7;11], *HOXD13* [t 2;11]); while non-HOX gene fusion products involve *MLLT10*, *DDX10* (inv 11), *KDMA5* (*JARID1A*, t 11;21) and *NSD1* (t 5;11), which is the most common fusion oncoprotein in pediatric AML. Frequently, partner proteins share their distinctive domains (PHD or SET domains) to synergize a chromatin remodeling action and/or transcriptional regulation: these effects led to an aberrant transcription of key genes (e.g., *MEIS1*). Downstream effects can also determinate self-renewal of HSPCS, block of differentiation, but also alter the formation of mitotic spindle and impair the mitotic checkpoint complex [18,19].

However, even in the largest study (1023 patients) on molecular characterization of pediatric AML, included in the TARGET initiative, only a relatively small group of primary chemoresistant patients was analyzed and only a subgroup of them (22 patients) can be strictly defined as PIF patients [9,11]. Although genetic heterogeneity was observed in the primary chemotherapy resistance group, the gene expression profiles were remarkably similar among three different genetic subgroups. Those results support the hypothesis that in pediatric PIF-AML a common gene expression program is set in motion by different somatic mutations [11] and that such gene expression profile is related to the mechanism of chemoresistance. The latter conclusion provides the rationale for the use of transcriptomic data generated in previous studies on genetically characterized pediatric AML [20] in order to corroborate and expand the results obtained in the TARGET study.

In the present study we tackled the identification of genes involved in PIF by leveraging on the observation that NUP98 gene-fusion products are enriched in PIF-AML pediatric patients [9,10,11]. Indeed, one of the primary chemoresistant genetic subgroups described by McNeer et al. [11] was characterized by the presence of NUP98r, and additional point mutations in few selected genes (including *WT1*, *ELF1* and *FRMD8*). The public availability of genome-wide transcriptome analysis in patients characterized for their primary response to therapy (PIF or PIR) and for the presence or absence of NUP-98 rearrangements (NUP98r+ or NUP98r−) [9] allowed us to identify NUP98r/PIF-associated genes and to validate their association to NUP98 rearrangements in a different set of transcriptomic data [20]. Moreover, we provide another independent validation of the association of the identified NUP98r associated gene expression changes to the PIF status in our local series of PIF and PIR pediatric AML patients by transcriptome arrays and quantitative real-time PCR analysis.

## 2. Results

### 2.1. NUP98 Rearrangements Frequency Is Comparable in Cohorts Belonging to Three Different Programs (AIEOP-AML Group; TARGET; Local Cohort)

**Figure 1:**
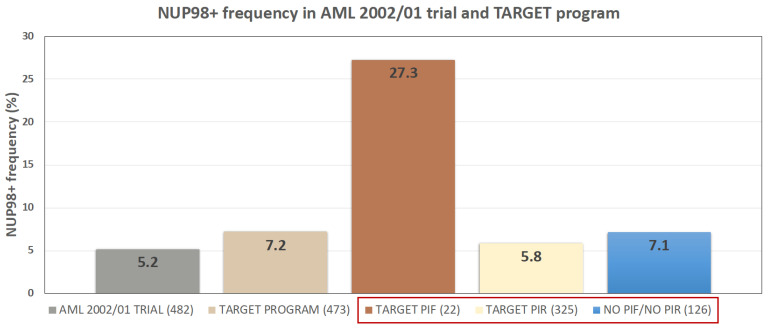
NUP98r frequency in AIEOP AML 2002/01 and TARGET program. The TARGET study distinguishes PIF from PIR patients. The three subgroups boxed in red are included in TARGET study.

To identify differential expressed genes (DEGs) in NUP98r+ versus NUP98r− patients, firstly we performed a frequency analysis of NUP98-rearrangements in two cohorts included in this study (AML 2002/01 trial and TARGET program). As described in Bisio et al. [20], 25 out of 482 patients enrolled to AML 2002/01 clinical trial showed NUP98 rearrangements (5.2%, Figure 1); among these, 85 samples were analyzed by expression microarray (Human Transcriptome Array 2.0 platform) and included 19 NUP98r+ samples (22.3%). In detail, 11/19 are NUP98-NSD1 (57.9%), 2/19 are NUP98-KDM5A (10.5%), 3/19 are NUP98-PHF23 (15.8%) and finally 3/19 are NUP98-HOXD13, NUP98-LEDGF and NUP98-DDX10 (15.8%). We compared these results with RNA Seq data obtained in the framework of the program called “Therapeutically Applicable Research to Generate Effective Treatments” (TARGET): 34 out of 473 analyzed samples are NUP98r+ (7.2%), of which 20 are NUP98-NSD1 (58.8%). NUP98-rearrangements are enriched in PIF group (6/22; 27.3%) compared to PIR (19/325; 5.8%), as recently reported [10,11]; the TARGET program also includes 126 patients who do not meet criteria for both PIF and PIR definition and then are excluded from the subsequent analysis (Figure 1).

### 2.2. Differentially Expressed Genes between NUP98r+ and NUP98r− Samples

**Figure 2:**
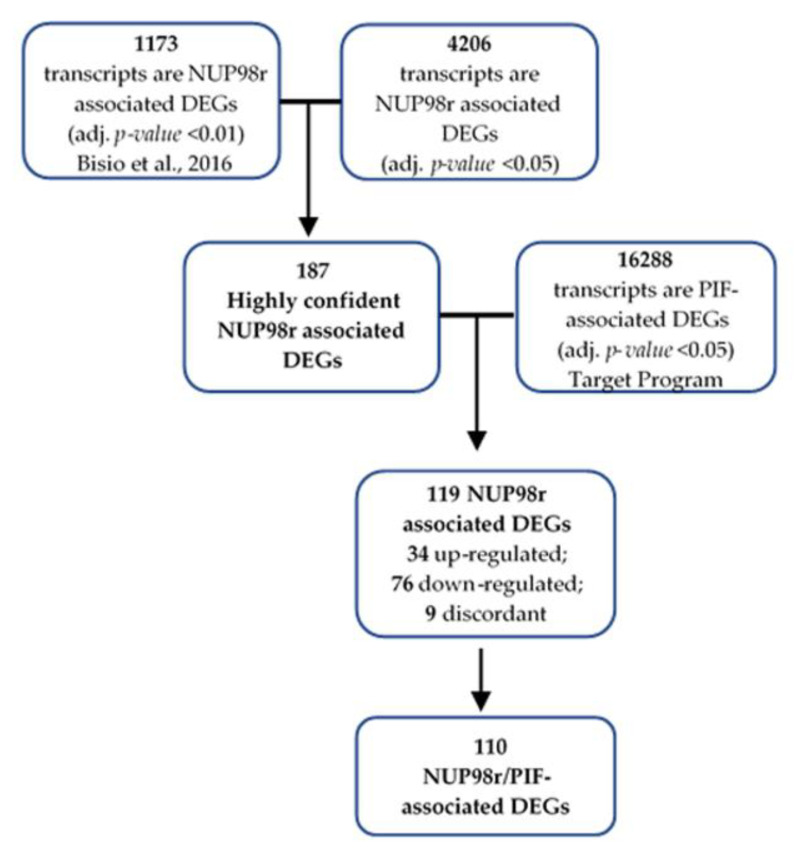
The workflow and overall steps in the integrated analysis in order to identify “NUP98r/PIF-associated DEGs”. The figure shows first 187 highly confident NUP98r associated DEGs, defined as the overlapping fraction between DEGs obtained by analyzing datasets of GSE75461 and TARGET program. Then, overlapping 110 NUP98r/PIF-associated DEGs was obtained.

In order to identify in a robust way a significant fraction of DEGs depicting the molecular signature of NUP98r, we compared the list of 1173 differentially expressed transcripts, containing 588 coding RNAs and 585 non-coding RNAs, obtained analyzing data from 19 NUP98+ patients and 66 NUP98r− patients, previously reported by Bisio et al. [20], with the list of 4206 differentially expressed transcripts (adjusted *p*-value < 0.05) obtained by analyzing RNA-Seq data from 21 NUP98+ and 137 NUP98r− patients, publicly available through the TARGET program (https://ocg.cancer.gov/programs/target/projects/acute-myeloid-leukemia accessed on 10 February 2020). Such comparison allowed us to identify 187 transcripts (158 coding and 29 non-coding RNAs) that are differentially expressed in both datasets (Figure 2). We called those 187 DEGs “highly confident NUP98r associated DEGs”. Most of them have similar behavior in both studies: 55/57 and 124/130 are similarly upregulated and downregulated, respectively. In particular, in both datasets we observed a strong concordant upregulation of some genes such as *DEFA1*, *HOXB5*, *DEFA4*, *HOXB6*, *CCNA1*, *NT5C3A* and *SPINK2*.

### 2.3. Primary Induction Failure-Associated DEGs Are Enriched among Highly Confident NUP98r Associated DEGs

By comparing gene expression values obtained in PIR and PIF patients of the Target study (Figure S1), we selected 16,288 transcripts differentially expressed (adjusted *p*-value < 0.05) between the two-groups (called PIF-associated DEGs; Figure 2 and Figure S1). Indeed, PIF-associated DEGs are significantly enriched in the list of 187 highly confident NUP98r associated DEGs (2.23-fold enrichment compared to expectations; hypergeometric *p*-value = 2.35e−23). The 119 transcripts (105 coding and 14 non-coding) shared among NUP98r associated DEGs and PIF-associated DEGs includes 34 upregulated and 76 downregulated transcripts both in PIF and NUP98r+ patients. Only nine transcripts were discordant between the two groups (Figure 2; Table S1) and were not further analyzed. Therefore, 110 concordant transcripts are called “NUP98r/PIF-associated DEGs” (Figure 2).

In order to establish which of these 110 DEGs have predictive diagnostic accuracy we evaluated each transcript for the diagnosis of PIF by ROC curve. Results of “Area Under the Curve” (AUC) and *p*-value are reported in Supplementary Table S2. In order to prioritize transcripts for further validation we selected 23 upregulated and 14 downregulated transcripts showing AUC > 0.85 and *p*-value < 0.001. As a validation set, we used a local cohort of 12 pediatric AML patients (PIF = 4; PIR = 8). Whole transcriptome analysis (HTA 2.0) was performed in samples derived from the local cohort in order to evaluate the expression status of 67,529 different genes. However, due to the relatively low number of patients, no transcript reached a significant *p*-value after adjustment to multiple comparisons. Therefore, this local set of transcriptomic data was used only to further select “candidate PIF diagnostic markers” (AUC > 0.85, *p*-value < 0.001) among the 110 “NUP98r/PIF-associated DEGs”. Only 9/23 upregulated transcripts show a concordant up-regulation (linear fold change ≥ 1.3) in the local cohort transcriptomic data (Table 1). Two transcripts, TRDMT1 and NDUFA5, were not further analyzed due to low gene expression values (Robust Multi-array Average, RMA < 5) in local cohort. In order to investigate whether the selected genes (*SPINK2*, *TMA7*, *CAPZA1*, *FGFR1OP2*, *MAN1A2*, *NT5C3A* and *SRP54,* Figure S3) might play a diagnostic role in adult PIF-AML, we analyzed publicly available RNAseq data obtained in an adult cohort (OHSU study) [4]. Moreover, two genes (*SPCS2* and *CDCP1*) belonging to the 34 upregulated NUP98r/PIF associated DEGs (Table S2) were added to analysis due to the high Positive Predictive Value (*SPCS2*) or previous studies on hematological tumors (*CDCP1*) [21,22].

**Figure 3:**
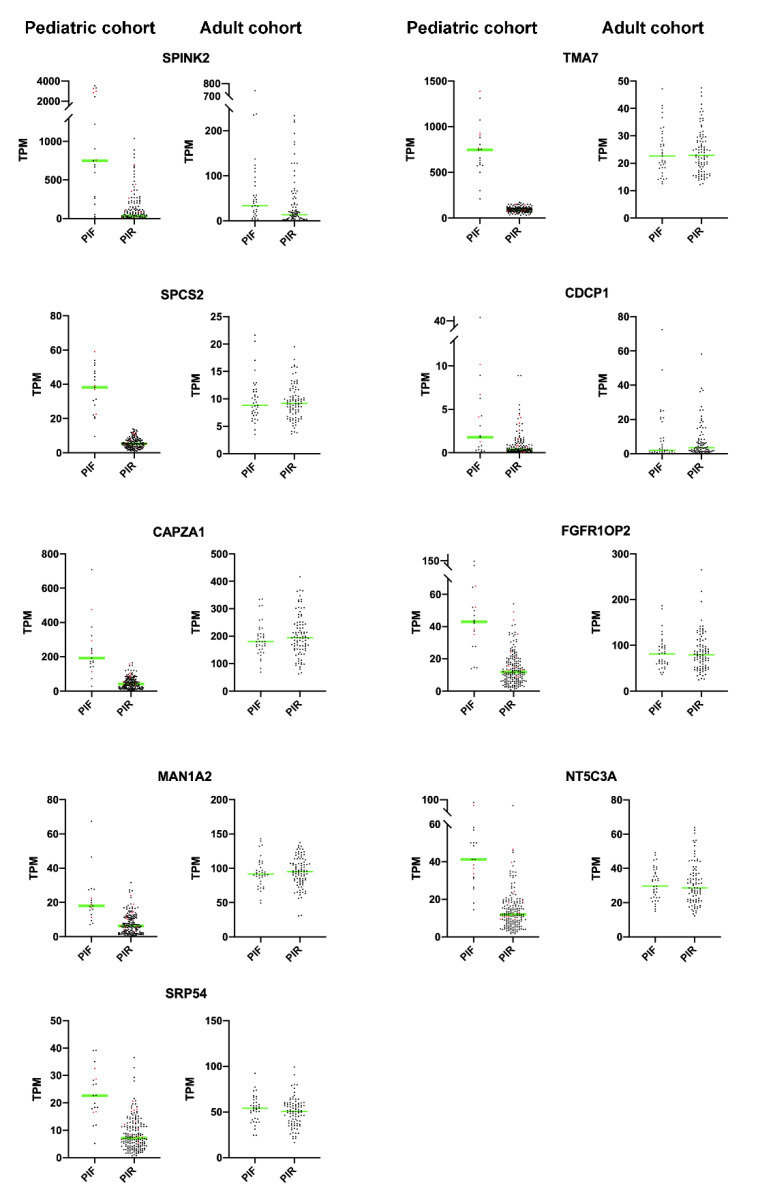
Dot-plots showing expression values for SPINK2, TMA7, SPCS2, CDCP1, CAPZA1, FGFR1OP2, MAN1A2, NT5C3A and SRP54 as TPM in pediatric and adult cohorts. NUP98r+ patients are showed in red dots. In adult patients a significant upregulation in PIF vs. PIR has been detected only for SPINK2 (t-test unpaired parametric *p*-value < 0.05); green line corresponds to mean value.

Figure 3 shows TPM values (dot plot) for both PIF and PIR patients in pediatric and adult cohorts: *SPINK2* is the only gene that confirmed its significant upregulation in adult PIFs (*n* = 37) vs. PIRs (*n* = 90) (*p*-value < 0.05). The difference between pediatric and adult cases is in agreement with the well-known observation that the range of mutations is different in adult vs. pediatric cases. It is possible that childhood PIF AML cases are more homogeneous and mechanistically different from adult cases.

We further investigated the expression of the selected genes on the “UALCAN portal”, a web-portal publicly available at http://ualcan.path.uab.edu (accessed on 10 February 2020), that permits to analyze relative expression of a query gene across tumors and normal tissues selected by “The Cancer Genome Atlas” datasets [23]. This analysis, accessible only for adult AML samples, revealed that SPINK2, CAPZA1, FGFR1OP2 and MAN1A2 are expressed at higher levels in AML dataset compared to 33 TCGA (The Cancer Genome Atlas) cancers with different histotypes, and the higher expression is observed in AML M0 (undifferentiated cells) based on FAB classification (Figure S2). The same analysis conducted on TMA7, SPCS2, CDCP1, NT5C3A and SRP54 revealed a homogeneous expression in the majority of 33 TCGA histotypes of cancer (Figure S2).

### 2.4. Validation by Quantitative Real-Time PCR in Local Cohort

**Figure 4:**
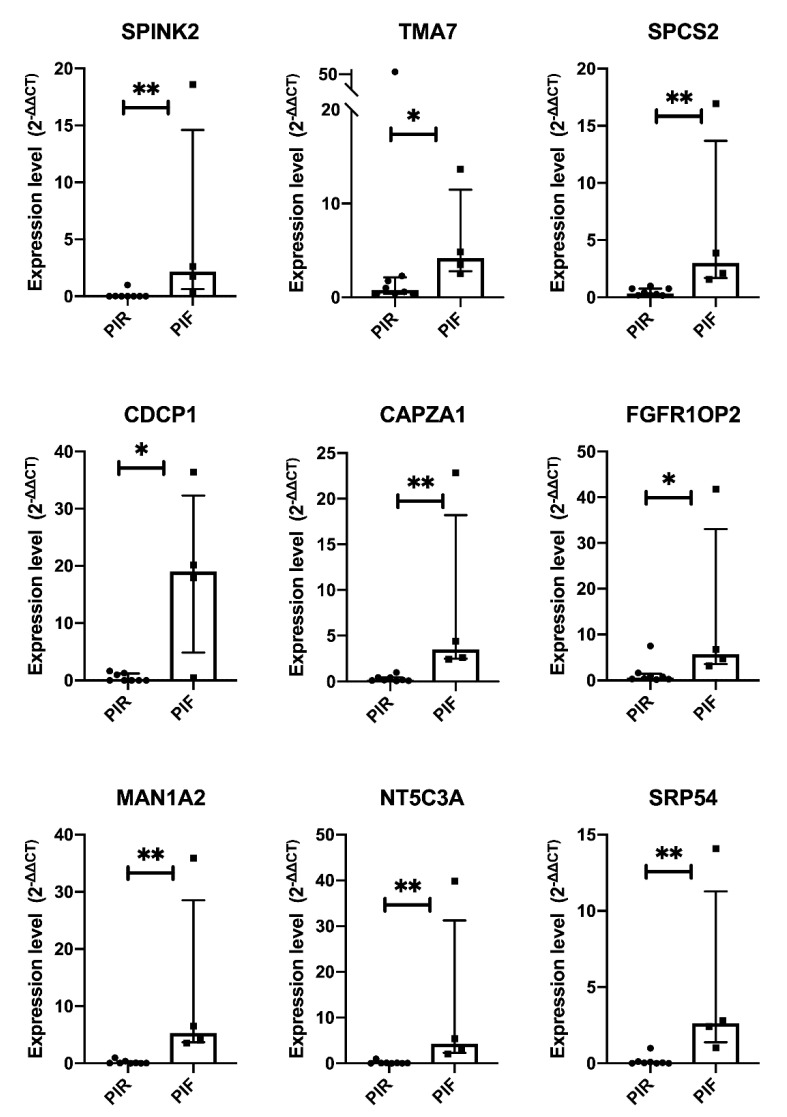
Quantitative Real Time RT-PCR of selected transcripts in local pediatric AML cohort. Statistical differences between PIF and PIR patients were assessed using the Mann-Whitney test *p*-value. * *p* < 0.05, ** *p* < 0.01.

By quantitative Real-Time PCR we evaluated the transcript levels of SPINK2, TMA7, SPCS2, CDCP1, CAPZA1 FGFR1OP2, MAN1A2, NT5C3A and SRP54 in our local cohort. As shown in Figure 4, we confirmed a significant upregulation for all tested transcripts in PIF patients compared to PIR (Mann-Whitney test *p*-value < 0.05).

### 2.5. NUP98-NSD1 and NUP98-KMD5A Comparison

**Figure 5:**
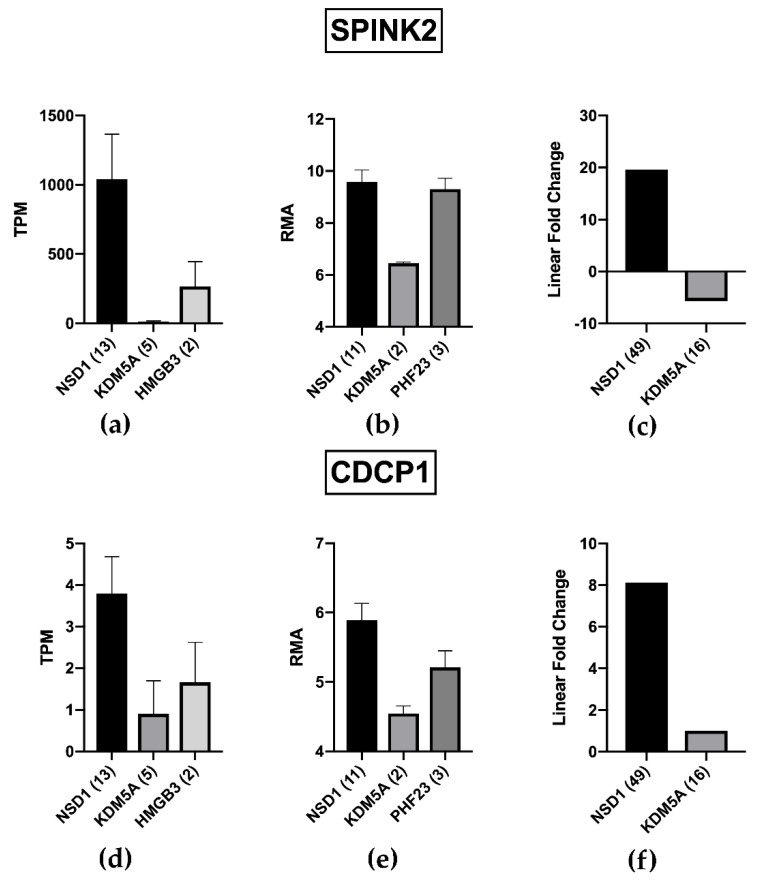
SPINK2 and CDCP1 are differentially expressed depending on NUP98r. (**a**,**d**) TARGET study shows a significant upregulation of SPINK2 and CDCP1 in presence of NUP98-NSD1 rearrangement, whilst in the first one it is almost absent in patients with NUP98-KSM5A fusion. (**b**,**e**) GSE75461 cohort establishes the higher expression of SPINK2 and CDCP1 in patients with NUP98-NSD1 compared with NUP98-KDMA5 rearrangements, revealing also a comparable expression of SPINK2 in presence of NUP98-PHF23 translocation. (**c**,**f**) Noort’s study confirms that SPINK2 and CDCP1 are dramatically upregulated in presence of NUP98-NSD1 rearrangement. For CDCP1 no significant difference in Fold Change between KDMA5+ and KDMA5− has been reported by Noort et al. We indicated a Fold Change = 1 to allow a visual comparison.

In order to further validate the obtained results, we exploited the recent availability of novel RNA-Seq data from the collaborative study between the Children’s Oncology Group (COG) and European AML study groups, aimed to define gene expression profile associated to NUP98-KDM5A and NUP98-NSD1 rearrangements in childhood AML. Those authors report 3026 statistically significant DEGs between AML with or without NUP98-NSD1 and 2177 between AML with or without NUP98-KDM5A. We found that 35.5% of our NUP98/PIF associated genes (39 genes out of 110) are present among the list of genes detected in NUP98-NSD1 group by Noort et al., [24] (8 upregulated and 31 down-regulated; Table S3). Interestingly, the list of upregulated genes includes SPINK2 and CDCP1, confirming their association to the specific NUP98-NSD1 rearrangement and PIF status. Indeed, SPINK2 and CDCP1 resulted dramatically decreased in samples bearing the NUP98/KMD5A fusion in three different studies (Figure 5), suggesting that the difference in their expression may contribute to the biological and clinical difference between the two NUP98 rearrangements.

### 2.6. HOXA and HOXB Clusters, MYC and CDK6 Expression

**Figure 6:**
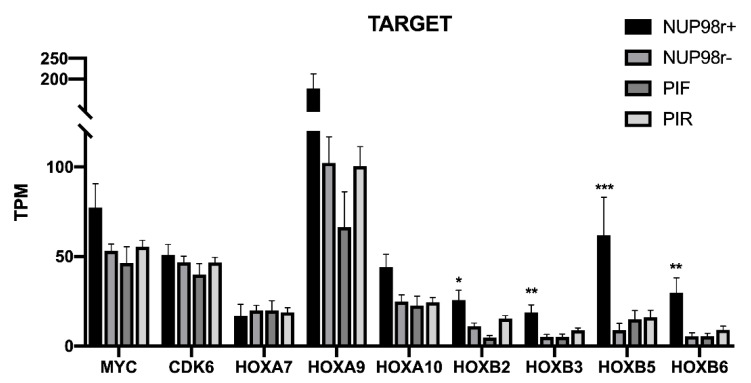
Expression values of MYC, CDK6, HOXA and HOXB cluster genes in TARGET cohort, subdivided in NUP98r+ (*n* = 21) and NUP98r− (*n* = 137) patients, PIF (*n* = 19) and PIR (*n* = 175) patients. Only HOXB cluster genes are significantly higher in NUP98r+ patients (*t*-test * *p* value < 0.01, ** *p* < 0.001, *** *p* < 0.0001), while no difference was detected comparing PIF and PIR patients.

It has been repeatedly shown that overexpression of MYC, CDK6 and several members of HOXA and HOXB clusters are associated with NUP98 fusions both in human AML and in animal models [14,25,26]. Moreover, recent studies have suggested that CDK6 is a direct transcriptional target of NUP98 fusion proteins [19]. With this in mind, we compared the expression of different HOXA and HOXB cluster genes, MYC and CDK6 between NUP98r+ and NUP98r− AML and PIF and PIR patients using the RNA-seq data of the Target cohort (Figure 6). Several HOXA and HOXB genes that have been reported as overexpressed in NUP98r+ in comparison to NUP98r− do not show a similar overexpression in PIF vs. PIR subgroups. CDK6 does not show significant differences among the analyzed groups, in agreement with its involvement in oncogenesis of different types of AML unrelated to NUP98 fusion proteins [19].

## 3. Discussion

One of the unsolved issues in the treatment of pediatric AML is the existence of a subgroup of patients that do not achieve complete remissions (PIF), even after two induction cycles, thus establishing a condition of primary chemotherapy resistance and failure of induction remission therapy. A better understanding of the molecular abnormalities underlying such condition may represent a crucial step towards the design of molecular tests for early prediction of the PIF condition and the development of specific treatments.

The strategy followed in our study for the identification of PIF-associated genes is based on the observation that NUP98 gene-fusion products are enriched in PIF-AML pediatric patients (Figure 1) [9,10,11,20]. The role of NUP98 in the induction failure is not fully understood because several AML patients bearing NUP98r undergo complete remissions (CRs). It is likely that NUP98 rearrangements must be associated with other molecular alterations and such association leads to induction resistance and low survival [10].

Moreover, NUP98 can be fused with different gene partners, such as *NSD1*, *KMD5A*, and *PHF23* [18]. Indeed, it has been recently shown that NUP98-NSD1 and NUP98-KMD5A present substantial gene expression difference [24]. NUP98-KMD5A is an independent risk factor for poor overall survival and relapse [24] but, in contrast to what has been repeatedly shown for NUP98-NSD1 [10], it does not significantly influence the probability of complete remission after induction [24]. However, an increased percentage of patients with minimal residual disease has been reported among those bearing NUP98-KMD5A [24].

On the basis of previous data, we hypothesized that a subgroup of genes overexpressed in NUP98 positive AML patients (and in particular in NUP98-NSD1 positive patients) could play a relevant role in the characterization of the PIF condition. Indeed, as a first step we generated a robust list of NUP98r associated transcripts, obtained combining the results of two different studies on pediatric AML [9,20]. These two studies analyzed the whole transcriptome using different techniques (microarray and RNAseq) and the relatively low number (187) of shared NUP98-associated transcripts is not surprising. However, our data show that this list of NUP-98 associated genes is highly enriched in PIF-associated genes, thus confirming the functional relationship between the gene-rearrangement and the clinical response to therapy. Indeed, the large majority of NUP98 rearrangements in the analysis reported by Bisio et al. [20] are NUP98-NSD1 (11 out of 19); in the TARGET pediatric AML study [9] as well, the only NUP98r observed in PIF patients is NUP98-NSD1. Although NUP98-NSD1 are more frequent among PIF patients, several PIF patients do not bear such gene fusion, and several patients bearing it obtain complete remission after induction therapy. Therefore, gene expression changes associate to NUP98-NSD1 may represent only a predisposing condition that requires the association of other molecular alteration to generate a full primary chemoresistance [9,10,11]. However, the identification of gene expression dysregulations in both groups (PIF and NUP98- bearing cells) may pave the way towards the identification of critical steps in the generation of the primary chemoresistant phenotype. Moreover, the validation provided by the comparison of different datasets may increase the robustness of the data. Indeed, by comparing results from three different studies [9,20,24] we confirm the upregulation of two genes, *SPINK2* and *CDCP1*, in pediatric PIF-AML patient bearing the NUP98/NSD1 rearrangement. Moreover, expression of SPINK2 and CDCP1 were higher in pediatric AML with NUP98/NSD1 in comparison to those with NUP98/KMD5A (Figure 5), suggesting that the differential expression of this gene can contribute to the higher primary chemoresistance observed in NUP98/NSD1 positive patients. Indeed, Ostronoff et al. [10] reported that the complete remission rate for NUP98-NSD1 positive and negative patients was 50% and 77%, respectively. Such poor response to primary induction was not observed in the case of NUP98-KDM5A: Noort et al. [24] did not report significant difference in complete remission rates between NUP98-KDM5A positive and negative patients (91% vs. 90%, respectively). An interesting hypothesis is that the differential expression of SPINK2 and CDCP1, observed in the present study, could play a role in the different clinical outcome observed between NUP98-NSD1 and NUIP98-KMD5A rearrangements.

We attempted the first validation of the identified NUP98r/PIF associated genes in an independent cohort of PIF and PIR pediatric AML patients. However, our single-center study had the availability of a rather limited number of PIF samples, since pediatric AML is a rare disease and PIF represent only a low percentage of total cases (10–15%) [3]. The relatively low number of patients can be considered one of the limitations of our study. Nevertheless, we were able to confirm the differential expression of nine genes belonging to the list of NUP98r + /PIF-associated genes in our local cohort, thus suggesting that those selected transcripts may represent good candidates for biomarker validations in larger multicenter studies. In agreement with the strategy used for identification, we evaluated those biomarkers at the transcript level. However, an analysis at the protein level may provide further validation of their diagnostic utility and knowledge on their biological role. Indeed, CDCP1 is also known as CD318 and flow cytometric evaluation of this membrane protein on adult AML cells has been already reported [27]. Another limitation of the study was the unknown level of heterogeneity of the PIF status in pediatric AML. A high level of heterogeneity might have hampered the identification of important subgroup-specific biomarkers. However, the design of larger multi-center studies can address such difficulties.

Interestingly, SPINK2 (Serine protease inhibitor Kazal-type 2) is the most altered among all the upregulated genes in PIF patients compared with PIR. It is known as trypsin-acrosin inhibitor [28] and was firstly identified in human spermatozoa. In 2009, Ting Chen et al. reported its strong expression in different cell lines particularly in Daudi (Burkitt’s lymphoma) and HL-60 (myelocytic leukemia) model lines, and correlated its role to tumor expression and treatment response [29]. Regarding its function the researchers reported the SPINK2 3D structure and demonstrated its protease inhibitor activity on trypsin [29]. To date its role in the physiology of myeloid and lymphoid cells and the functional meaning of its overexpression in cancer remains to be elucidated. Recently Xue et al., analyzing in silico data deposited in Oncomine (cancer microarray database), reported an higher expression level of SPINK2 in 542 AML vs. 74 normal samples and 9 AML vs. 3 PBMCs of normal samples, respectively; this result was further confirmed in Gene Expression Profiling Interactive Analysis (GEPIA, http://gepia.cancer-pku.cn/, accessed on 10 February 2020) database, including 173 AML vs. 70 healthy individuals; moreover the authors, using Ualcan (http://ualcan.path.uab.edu/analysis.html, accessed on 10 February 2020), have shown that SPINK2 upregulation is correlated with poor prognosis of AML patients, belonging to TCGA dataset. Then the authors validated SPINK2 overexpression in 12 AML adult patients by quantitative q-PCR, ultimately concluding that SPINK2 could play a key role in AML development [30]. SPINK2 upregulation was also found in Diffuse Large B-cell Lymphoma [31] and Primary Cutaneous Follicle Center Cell Lymphoma samples [32].

Another differentially expressed transcript, that is robustly associated to NUP98/NSD1 fusion and to PIF status, is CDCP1 (CUB-Domain Containing Protein 1), also known as SIMA135, gp140, CD318 and Trask, which is upregulated in other malignancies including breast, colon and lung cancers, but only few studies evaluated its expression and role in hematological tumors [33]. In hematopoietic system, CDCP1 is expressed in CD34+ stem cells but not in differentiated cells [21]. This observation has been extended by Büring et al., who analyzed leukemic blasts from patients with ALL, AML or CML, showing that CDCP1 is expressed in CD34+/CD38− and CD34+/CD133+ cells, but not in mature erythroid progenitors; moreover, it is expressed at similar levels as CD133, especially in AML blasts [22]. The authors suggest that CDCP1 could be used as independent marker for the diagnosis of leukemia, concluding that it appear to characterize immature erythroid and B lymphoid precursor cell subset [22]. Promoter region of the coding gene for *CDCP1* is highly rich of CpG island, whose methylation status drives the expression pattern of *CDCP1* itself in cancer; this can justify the detection, until 2006, of this transcript product only in K562, considered as the only CDCP1- hematopoietic positive cell lines, which derived from the blast crisis of chronic myeloid leukemia (CML) [34]. Furthermore, it has been demonstrated that CDCP1 is overexpressed in CD34+ positive cells of Nilotinib-resistant CML patients with t(9;22), suggesting that CDCP1 can prevent Nilotinib-induced cell death, by cooperating with PKCδ, with its multiple attachment sites [35]. Recently, it has been shown that the overexpression of CDCP1 in AML patients, receiving high dose of anthracycline within the induction therapy, constitutes a negative prognostic factor, generally more expressed in undifferentiated AML phenotypes (M0, M1) [27].

Even if only SPINK2 and CDCP1 were confirmed in three cohorts, seven other genes successfully passed the validation phases crossing GSE75461, TARGET and local cohort data. Among these, TMA7 (Translation Machinery Associated 7 Homolog) and SPCS2 (Signal Peptidase Complex Subunit 2) reached the highest levels of diagnostic accuracy in our study, but their role in cancer is still unknown. CAPZA1 (Capping Actin Protein Of Muscle Z-Line Subunit Alpha 1) is member of the F-actin capping protein alpha subunit family, which acts into the cytoskeleton binding to the barbed ends of actin filaments and mediating the actin polymerization [36,37]. Recently, Huang et al. demonstrated that CAPZA1 reduces epithelial-mesenchymal transition in hepatocellular carcinoma, preventing migration and invasion [36], likewise in gastric cancer [38]. FGFR1OP2 (Fibroblast Growth Factor Receptor 1 Oncogene Partner 2) encodes for a protein, called FOP2, characterized by four putative coiled-coil domains able to dimerize and oligomerize. In fibroblasts it seems to be associated with cytoskeleton’s fibers and to mediate the contraction of the collagen gel, playing a role in cell motility [39].This gene is involved in the formation of chimeric gene *FGFR1OP2-FGFR1* (fibroblast growth factor receptor 1), that codify for a protein with a constitute tyrosine kinase activity, probably due to the presence of coiled-coil domain [40]. Furthermore, using UALCAN tool, FGFR1OP2 appears to be positively correlated to SPINK2 (data not shown). MAN1A2 (Mannosidase Alpha Class 1A Member 1) belongs to a family of a key enzymes for mature glycosylation into the Golgi complex. [41]. Although the involvement of α-1,2 mannosidases has been reported in cancer, their role is still unclear: MAN1A2 has been included among prognostic indicators for to B cell lymphoma [42]. NT5C3A (cytosolic 5′-nucleotidase-III), a cytosolic member of enzyme family (5′-nucleotidase family) that promotes dephosphorylation of nucleoside monophosphates to nucleosides and orthophosphate; in particular, the NT5C3 proteins catalyzes the dephosphorylation of pyrimidine analogues. Regarding its role in cytarabine pathway Li et al. showed the association of NT5C3 with cytarabine/gemcitabine cytotoxicity [43], while Cheong et al. suggested that a single nucleotide polymorphism of *NT5C3* gene may discriminates responsive patients after induction phase [44]. SRP54 (Signal Recognition Particle 54) is a part of signal recognition particle (SRP), a ribonucleoprotein, that mediates the passage between the ribosomes and membrane associated protein-translocation machinery of the endoplasmic reticulum (ER). Recently, Goldberg et al. reported a novel mutation (in frame deletion) that affects complex stability causing congenital neutropenia [45], as described also in previous studies [46,47].

In conclusion, our findings provide a better knowledge of primary induction failure in pediatric AML, suggesting that upregulation of a specific group of genes, linked to NUP98r (in particular NUP98-NSD1), led to a primary chemoresistance and worse prognosis. Based on previous data, we suggest a synergic cooperation of selected NUP98/PIF-associated DEGs, which involves SPINK2, a serine protease inhibitor that could play a pivotal role in the maintenance of undifferentiated cell state CD34+ like cell state, and CDCP1, a membrane protein regulating growth, proliferation and invasion via Src, PKCδ and metalloproteinases activity.

## 4. Materials and Methods

### 4.1. Patients

The main clinical and pathological features and molecular alterations of AIEOP LAML cohort and TARGET cohort are reported in Supplementary Table S4. Moreover, the same characteristics for selected TARGET samples, analyzed by transcriptome analysis and subdivided in NUP98r positive and NUP98r negative patients, are reported in Supplementary Table S5. The effect of NUP98-rearrangements on survival is shown in Supplementary Figure S4 for the entire cohort of TARGET samples or for selected samples analyzed by transcriptome analysis. NUP98-rearrangements are associated to a poor prognosis in both analyses, but the difference reached a statistical significance level only in data deriving from the entire cohort (Figure S4).

The local pediatric cohort (*n* = 12; age, range 0–18 years, median age at diagnosis = 12; 4 males and 8 females, Table S4) consists of children, diagnosed and treated in our Center from 2013 to 2016, with available frozen bone marrow (BM) samples taken at diagnosis and provided by the Center of Pediatric Hematology Oncology (Azienda Policlinico University Hospital, Catania, Italy). The study was conducted in accordance with the Declaration of Helsinki and the protocol was approved by the Institutional Review Board of local IRB (n°71/2015/PO on date 15 June 2015). Four of these patients failed to achieve complete remission (CR) after two cycles of induction therapy (ICE), reporting all but one a blast rate between 10–20% (one case showed complete blast regeneration, major than 60%), detected by morphology and confirmed by flow-cytometry. Thus, these cases have been considered as Primary Induction Failure (PIF) patients.

The local control cohort includes eight patients who achieved CR after the same induction cycles of chemotherapy, and they were defined as Primary Induction Response (PIR) cases. In the attempt to perform a homogeneous analysis, we selected these cases as very-high-risk (VHR) patients, based on the genetic alterations detected at diagnosis: 2 with FLT3-ITD and NUP98-NSD1 fusion genes; 2 with FLT3-ITD and complex karyotype; 2 with DEK-CAN fusion genes; 2 with NUP98-translocations and complex karyotype (2018 WHO classification).

### 4.2. Human Transcriptome Array 2.0

RNA from leukemic blasts was extracted using the commercial RNeasy Mini Kit (cat. no. 74104, Qiagen, Milan, Italy) according to the manufacturer’s recommendations and total RNA was quantified by the NanoDrop spectrophotometer. As described previously [48,49], the array expression analysis was performed using 100 ng of total RNA to amplify and label targets in sense orientation for hybridization to the “Gene-Chip Human Transcriptome Array 2.0” according to manufacturer’s protocol (Cat. No. 902310, Cat. No. 900720; Affymetrix UK Ltd., High Wycombe, UK). Array scanning and data analysis were achieved through AFFYMETRIX^®^ EXPRESSION CONSOLE software (v 1.4, Affymetrix UK Ltd., High Wycombe, UK), that enables probe set summarization and initial data quality control, creates probe set intensity files (*.CEL) and probe level files using specific algorithm. Data have been further elaborated using AFFYMETRIX^®^ TRANSCRIPTOME ANALYSIS CONSOLETM (TAC) Software (Affymetrix UK Ltd., High Wycombe, UK), which performs statistical analysis and identifies differentially expressed genes. Data were submitted on GEO dataset (GEO, GSE163643).

### 4.3. Gene Expression Profiling Dataset

A larger expression array cohort, including 85 pediatric patients, with de novo AML, enrolled in the AIEOP AML 2002/01 trial (*n* = 482, 262 males and 220 females) was analyzed [3]; CELL and CHP files are available on Gene Expression Omnibus (GEO, GSE75461) [50]. DEGs genes were then confirmed within the pediatric AML cohort obtained from TARGET program [9]. Briefly it contains data from 1023 children enrolled in Children’s Oncology Group (COG) AML trials, whose clinical details and annotations are available through the TARGET Data Matrix (https://ocg.cancer.gov/programs/target/data-matrix, accessed on 10 February 2020). RNAseq data were downloaded as counts, analyzed by EdgeR software and log2 Fold-Change converted in linear Fold-Change, as we previously described [51]. From 358 samples available for mRNA sequencing analysis, we selected only bone marrow specimens for each patient (*n* = 242); then we divided them in NUP98-r positive (*n* = 21, median age = 9.3; 15 males and 6 females) and NUP98-r negative (*n* = 137, median age = 9.4; 70 males and 67 females) samples. To further investigate if NUP98-associated DEGs are enriched in PIF category, we selected patients who did not achieve complete remission after the first and second cycle of induction (PIF = 19, median age = 9.5; 12 males and 7 females) and those who achieved complete remission (Primary Induction Response, PIR = 175, median age = 9.7; 91 males and 84 females, Figure S1, Table S4). Further, we used the RNA data of adult AML deposited by Tyner et al. [4], which include 90 PIR (median age = 61; 44 males and 46 females) and 37 PIF samples (median age = 61; 24 males and 13 females). All clinical details, including therapy response, and molecular data are available as supplementary information (Table S4).

### 4.4. RNA Extraction and Quantitative PCR

From RNA extracted as reported above, reverse transcription was performed using 2 μg of total RNA, SuperScriptTM II Reverse Transcriptase (Cat. No 18064022; Invitrogen, Monza, Italy) and random primer hexamer. Primers were designed by the “Primers-BLAST” tool from NCBI (https://www.ncbi.nlm.nih.gov/tools/primer-blast/ accessed on date 10 June 2020); in Table S6 are reported for each transcript forward and reverse sequence primers, annealing temperature and fragment size. Quantitative real-time PCR analysis was performed using StepOne™ Real-Time PCR System by Applied Biosystems (Applied Biosystems, Foster City, CA, USA), on PIF and PIR samples. Briefly, the reaction (25 μL) was performed using 200 ng of cDNA. Each sample was analyzed in triplicate and the average was normalized to human MALAT1 expression. Amplification conditions included a cycle at 95 °C for 10 min followed by 40 cycles at 95 °C for 15 s and 56–61 °C for 1 min. As a negative control, reaction without cDNA was performed (no template control, NTC). The relative RNA expression level for each sample was calculated using the 2^−∆∆CT^ method, as previously reported [52].

### 4.5. Statistical Analysis

Statistical analysis on quantitative Real-Time experiments was conducted using GraphPad Prism 6 (v.6; GraphPad Software Inc., La Jolla, CA, USA). *p*-value, calculated using the nonparametric unpaired two-tailed Mann Whitney test, was considered significant with a value less than 0.05.

## Figures and Tables

**Table 1:** Linear Fold Changes between PIF and PIR patients were calculated using HTA 2.0 data in local cohort. Areas under Curve (AUC) and relative *p*-values were calculated using TARGET data. TRDMT1 and NDUFA5 (indicated in grey letters) were not further analyzed owing to low RMA values (<5).

Gene Symbol	Linear Fold Change (PIF vs. PIR)	AUC	*p*-Value *AUC*
***SPINK2***	1.96	0.89	<0.0001
***TMA7***	1.51	1.00	<0.0001
***CAPZA1***	16.13	0.95	<0.0001
***TRDMT1***	1.48	0.94	<0.0001
***FGFR1OP2***	2.86	0.92	<0.0001
***NDUFA5***	1.39	0.93	<0.0001
***MAN1A2***	3.08	0.89	<0.0001
***NT5C3A***	2.09	0.93	<0.0001
***SRP54***	2.73	0.91	<0.0001

## Data Availability

Local cohort data are available on GEO dataset (GEO, GSE163643).

## Supplementary Materials

The following are available online at https://www.mdpi.com/article/10.3390/ijms22094575/s1, Figure S1: Workflow about samples selection and their distribution in TARGET program and AML 2002/01 trial-GSE75461; Figure S2: UALCAN analysis on SPINK2, TMA7, SPCS2, CDCP1, CAPZA1, FGFR1OP2, MAN1A2, NT5C3A and SRP54 showing the expression levels in FAB subtypes and 33 different types of TCGA tumors; Figure S3: Roc Curve for SPINK2, TMA7, SPCS2, CDCP1, CAPZA1, FGFR1OP2, MAN1A2, NT5C3A and SRP54 for pediatric and adult cohort; Figure S4: Kaplan Meier curves showing the effect on Overall Survival of NUP98-rearrangements. Table S1: NUP98/PIF-associated DEGs; Table S2: Sensitivity, Specificity, Positive Predictive Value (PPV) and Negative Predictive Value (NPV) calculated for all 110 NUP98/PIF-associated DEGs; Table S3: List of thirty-nine DEGs obtained by crossing TARGET and Noort studies; Table S4: Characteristics of patients with AML included in our study; Table S5: Clinical and pathological features and molecular alterations of selected TARGET samples, analyzed by transcriptome analysis and subdivided in NUP98r positive and NUP98r negative patients; Table S6: Primers details used for qRT-PCR.
