# Supplementary material for: NUP-98 Rearrangements Led to the Identification of Candidate Biomarkers for Primary Induction Failure in Pediatric Acute Myeloid Leukemia
Source: Int J Mol Sci. 2021 Apr 27;22(9):4575. doi: 10.3390/ijms22094575 (PMC8123909; doi:10.3390/ijms22094575)
Supplement: Supplementary file 1 [file ijms-22-04575-s001.zip › 02 Figures Supplementary AMLPIF FINAL apr 22 21.pdf]

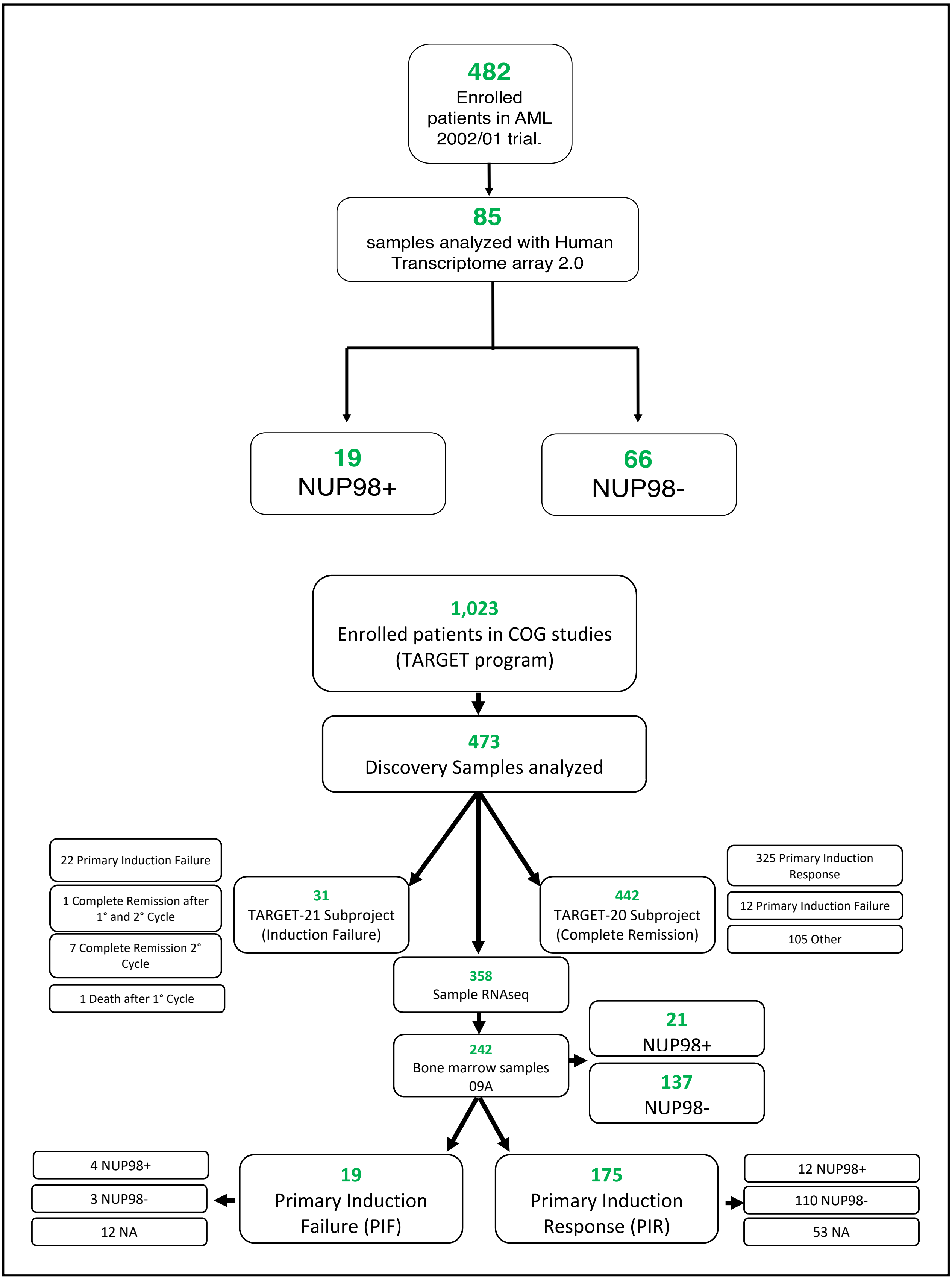

Figure S1. **Workflow about samples selection and their distribution in AML 2002/01 trial-GSE75461 and TARGET program.**

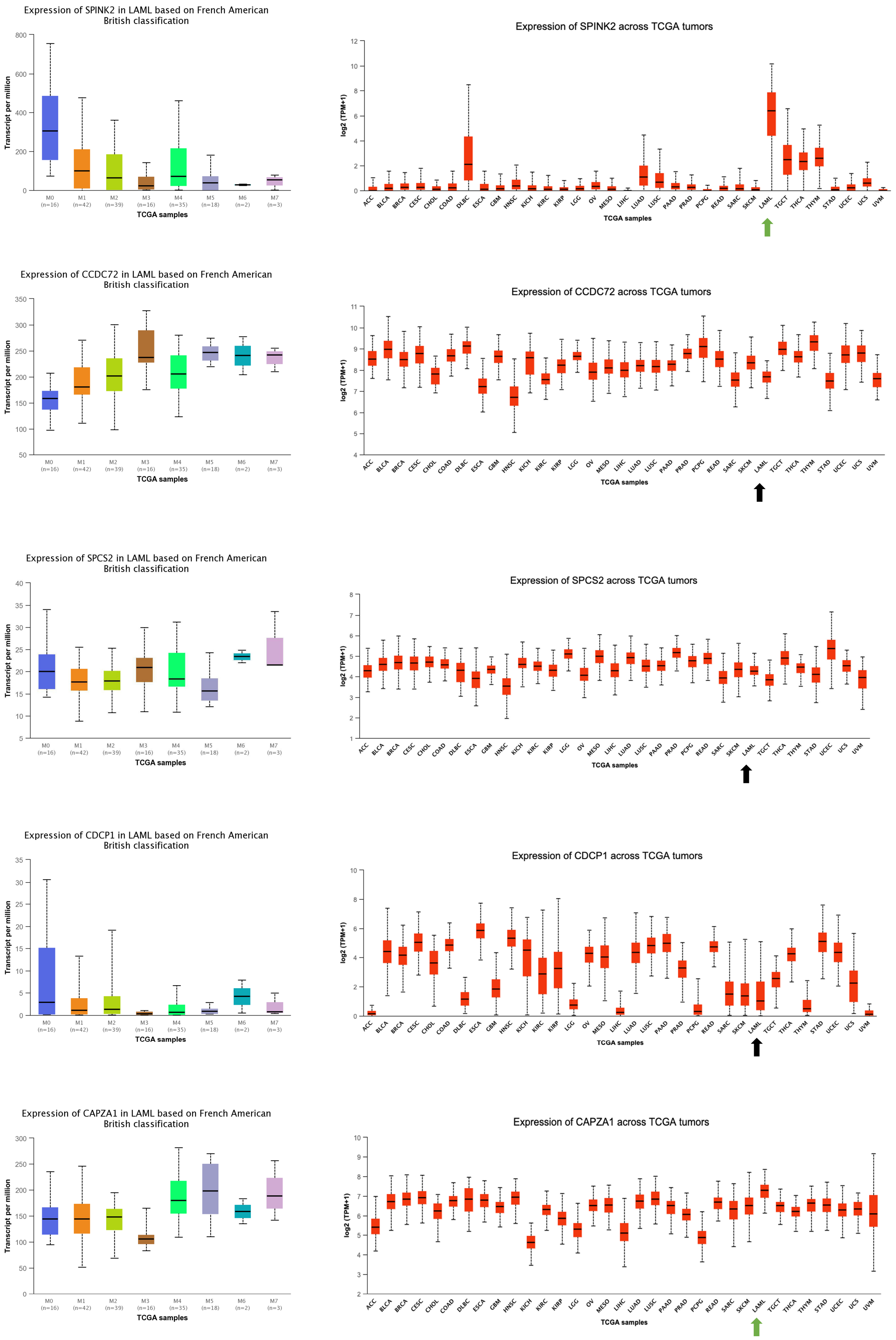
Expression of SPINK2 in LAML based on French American British classification
Transcript per million
M0 (n=16)
M1 (n=42)
M2 (n=39)
M3 (n=16)
M4 (n=35)
M5 (n=18)
M6 (n=2)
M7 (n=3)
TCGA samples
Expression of SPINK2 across TCGA tumors
log2 (TPM+1)
ACC BLCA BRCA CESC CHOL COAD DLBC ESCA GBM HNSC KICH KIRC KIRP LGG OV MESO LIHC LUAD LUSC PAAD PRAD PCPG READ SARC SKCM LAML TGCT THCA THYM STAD UCEC UCS UVM
TCGA samples
Expression of CCDC72 in LAML based on French American British classification
Transcript per million
TCGA samples
Expression of CCDC72 across TCGA tumors
log2 (TPM+1)
TCGA samples
Expression of SPCS2 in LAML based on French American British classification
Transcript per million
TCGA samples
Expression of SPCS2 across TCGA tumors
log2 (TPM+1)
TCGA samples
Expression of CDCP1 in LAML based on French American British classification
Transcript per million
TCGA samples
Expression of CDCP1 across TCGA tumors
log2 (TPM+1)
TCGA samples
Expression of CAPZA1 in LAML based on French American British classification
Transcript per million
TCGA samples
Expression of CAPZA1 across TCGA tumors
log2 (TPM+1)
TCGA samples

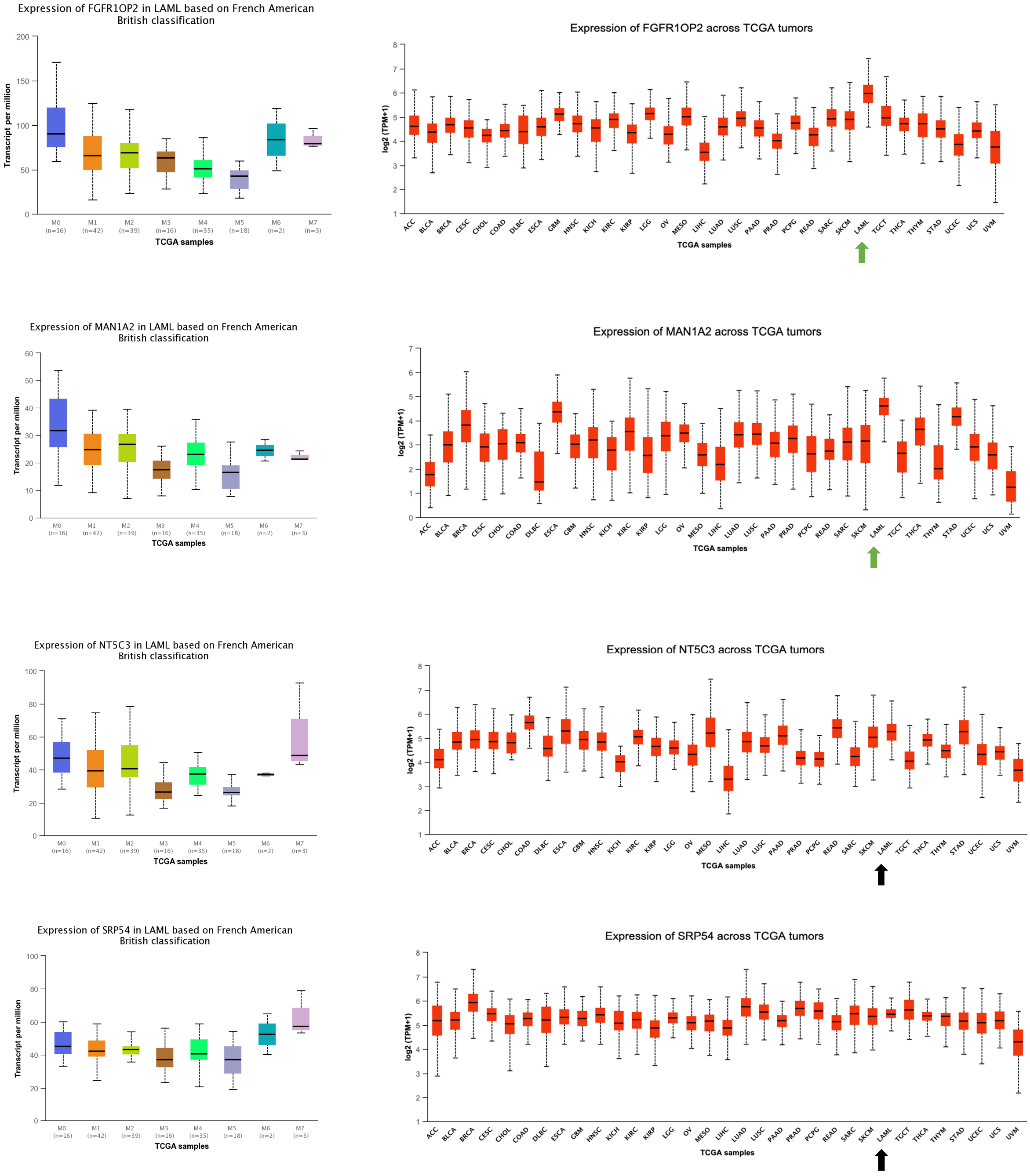

Figure S2. **UALCAN analysis on SPINK2, CCDC72 (TMA7), SPCS2, CDCP1, CAPZA1, FGFR1OP2, MAN1A2, NT5C3A and SRP54 showing the expression levels in FAB subtypes and 33 different types of TCGA tumors.** Arrows indicate AML hystotype; green arrows indicate higher expression in AML hystotype.

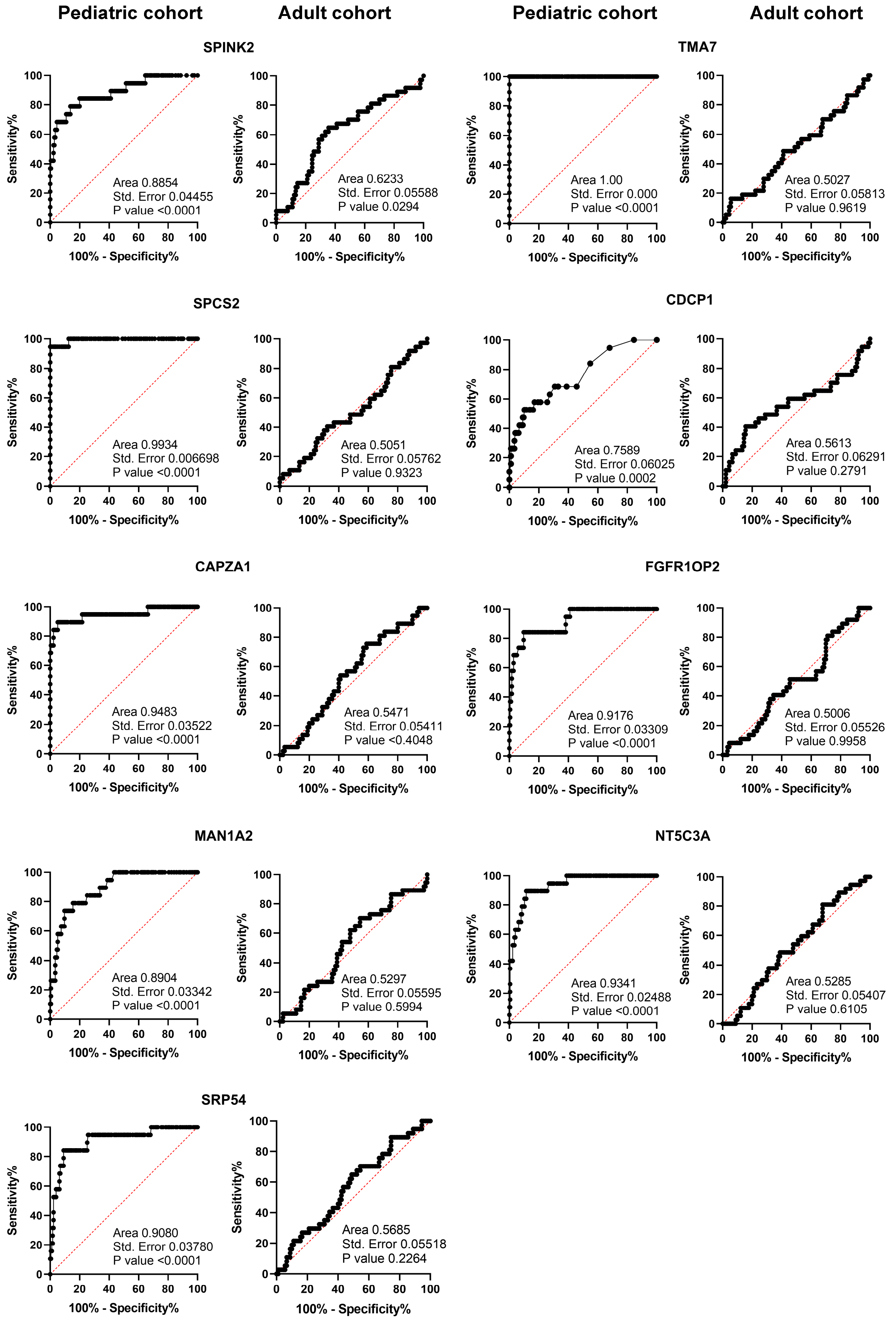

Figure S3. **Roc Curve for SPINK2, TMA7, SPCS2, CDCP1, CAPZA1, FGFR1OP2, MAN1A2, NT5C3A and SRP54 for pediatric and adult cohort.**

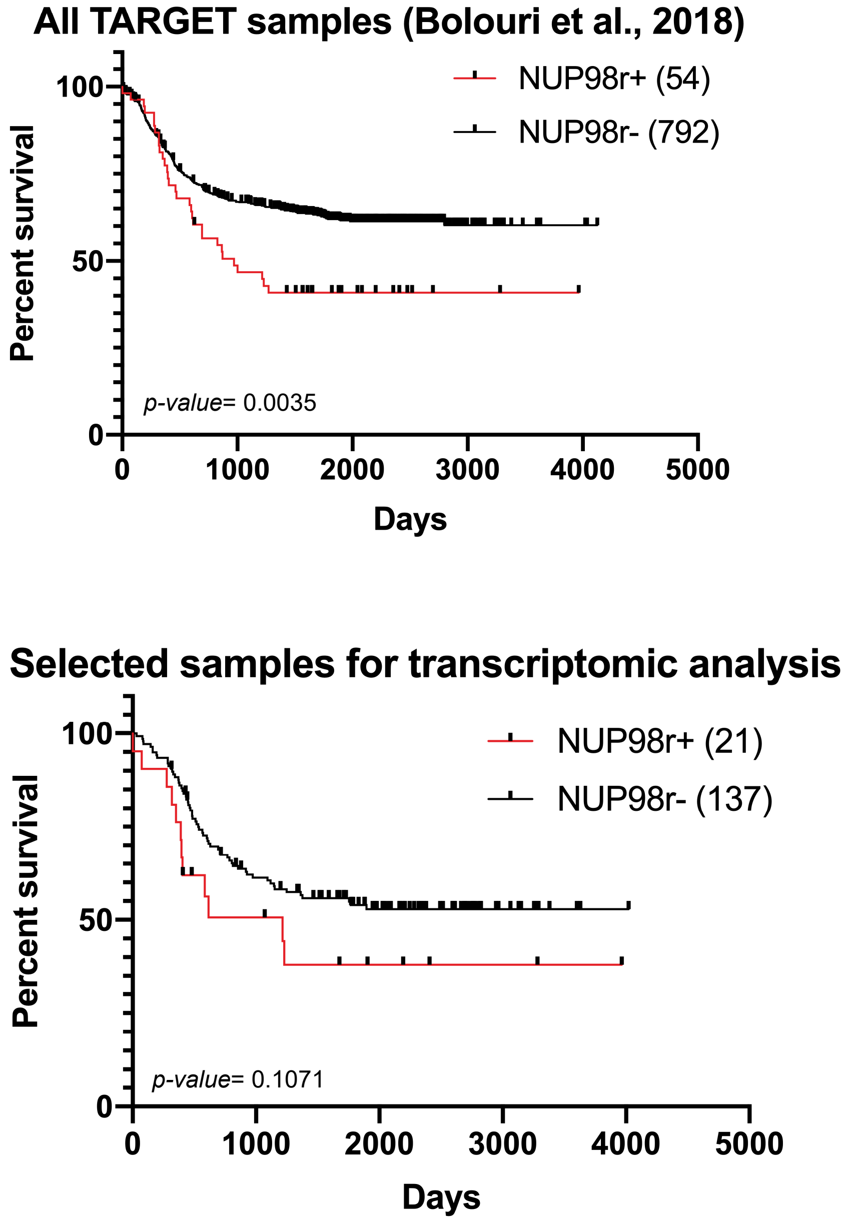

Figure S4. **Kaplan Meier curves showing the effect on Overall Survival of NUP98-rearrangements.** On the top, we show the effect of NUP98-rearrangements on overall survival in the entire cohort of TARGET program; on the bottom, the overall survival was calculated only in selected samples of TARGET program analyzed by transcriptome analysis.
