# Supplementary material for: NUP-98 Rearrangements Led to the Identification of Candidate Biomarkers for Primary Induction Failure in Pediatric Acute Myeloid Leukemia
Source: Int J Mol Sci. 2021 Apr 27;22(9):4575. doi: 10.3390/ijms22094575 (PMC8123909; doi:10.3390/ijms22094575)
Supplement: Supplementary file 1 [file ijms-22-04575-s001.zip › 03 Tables Supplementary AMLPIF FINAL apr 22 21.pdf]

34 up-regulated

	Gene name	Transcript type	Linear FC PIF (19) vs PIR (175)	Linear FC NUP98+ (21) vs Other AML (137)
1	RNU7-115P	snRNA	22.1	2.5
2	AC092171.1	antisense	9.7	2.5
3	TRGV2	TR_V_gene	9.3	4.1
4	SPINK2	Coding	8.3	4.7
5	CCDC144CP	pseudogene	5.5	1.9
6	AP002812.2	antisense	4.6	2.1
7	TMA7	Coding	4.6	1.6
8	DEFA4	Coding	4.3	5.8
9	SPCS2	Coding	4.2	1.5
10	CDCP1	Coding	4.0	2.4
11	ANP32E	Coding	4.0	2.2
12	LINC01881	pseudogene	4.0	1.5
13	AC026124.1	sense intronic	3.6	2.5
14	CAPZA1	Coding	3.2	1.5
15	NT5C3AP1	pseudogene	3.0	1.6
16	PAFAH1B2	Coding	2.8	1.6
17	TRDMT1	Coding	2.7	1.6
18	LGALS12	Coding	2.6	2.1
19	CCR6	Coding	2.6	3.0
20	NRG4	Coding	2.4	3.6
21	GNB5	Coding	2.2	2.1
22	FGFR1OP2	Coding	2.2	1.5
23	TMEM241	Coding	2.2	1.6
24	PCNT	Coding	2.2	1.5
25	NDUFA5	Coding	2.1	1.4
26	MAN1A2	Coding	2.1	1.6
27	GCNT1	Coding	2.1	2.0
28	NT5C3A	Coding	2.0	1.4
29	C9orf78	Coding	2.0	1.4
30	CPNE3	Coding	1.9	2.1
31	SRP54	Coding	1.8	1.4
32	CCDC25	Coding	1.7	1.6
33	CTR9	Coding	1.7	1.6
34	GOT1	Coding	1.4	1.7

76 down-regulated

	Gene name	Transcript type	Linear FC PIF (19) vs PIR (175)	Linear FC NUP98+ (21) vs Other AML (137)
1	GPR88	Coding	-54.0	-9.1
2	CLIP3	Coding	-51.8	-42.4
3	XPNPEP2	Coding	-20.5	-6.8
4	NA	Coding	-20.5	-16.9
5	FLT4	Coding	-20.1	-55.3
6	ALPK3	Coding	-19.6	-5.2
7	HSPG2	Coding	-18.2	-2.9
8	VIT	Coding	-17.7	-41.4
9	RCOR2	Coding	-17.6	-10.4
10	SEPN1	Coding	-14.4	-1.7
11	PODXL2	Coding	-14.1	-2.6
12	PACS1	Coding	-11.5	-1.5
13	GLIS3	Coding	-11.1	-20.5
14	GPR12	Coding	-10.2	-66.5
15	CELSR3	Coding	-10.1	-2.2
16	AADAT	Coding	-9.5	-6.5
17	AC104809.2	processed transcript	-9.2	-15.2
18	LPAR5	Coding	-8.8	-5.8
19	EVC2	Coding	-7.9	-17.9
20	ABCD1	Coding	-7.5	-2.1
21	GRAMD1A	Coding	-7.3	-1.9
22	SLC24A3	Coding	-7.0	-4.1
23	LRP5	Coding	-6.7	-2.0
24	PAQR5	Coding	-6.4	-6.5
25	RD3	Coding	-6.2	-47.7
26	NTNG2	Coding	-6.2	-2.6
27	TRH	Coding	-5.9	-8.1
28	DCHS1	Coding	-5.8	-7.4
29	KIF26B	Coding	-5.8	-3.7
30	AC239798.1	lincRNA	-5.5	-4.6
31	FBLN2	Coding	-5.4	-2.9
32	ARHGEF17	Coding	-5.3	-4.3
33	STK32B	Coding	-5.2	-4.4
34	CAPN15	Coding	-5.2	-1.5
35	TSC2	Coding	-5.2	-1.4
36	MAP4K2	Coding	-5.2	-1.6
37	HDAC7	Coding	-5.0	-1.5
38	CENPV	Coding	-4.9	-2.2
39	WHRN (DFNB31)	Coding	-4.8	-7.4
40	CACNA2D2	Coding	-4.8	-9.7
41	KCTD15	Coding	-4.7	-2.6
42	SHB	Coding	-4.5	-3.0
43	MMP25-AS1	antisense	-4.3	-3.0
44	PALM	Coding	-4.3	-9.7
45	TNFRSF21	Coding	-4.1	-2.4
46	DAGLA	Coding	-4.1	-3.2
47	ROBO4	Coding	-4.1	-10.3
48	RNF217	Coding	-4.0	-3.1
49	VSIG4	Coding	-4.0	-18.0
50	ENPP3	Coding	-4.0	-5.9
51	RNF217-AS1	antisense	-4.0	-6.3
52	SAP25	Coding	-3.9	-1.6
53	DCLK2	Coding	-3.8	-5.1
54	ZNF704	Coding	-3.8	-5.0
55	HPGD	Coding	-3.7	-7.1
56	DNM1	Coding	-3.7	-3.5
57	MMP28	Coding	-3.6	-8.1
58	ZNF467	Coding	-3.3	-3.1
59	ATP2A1	Coding	-3.3	-2.5
60	ARHGEF40	Coding	-3.2	-2.1
61	TANC1	Coding	-3.2	-10.9
62	PKN1	Coding	-3.1	-1.3
63	LOXHD1	Coding	-3.1	-11.8
64	TRIO	Coding	-3.1	-1.8
65	KDM4B	Coding	-3.0	-1.6
66	TRAK1	Coding	-3.0	-1.4
67	USP2	Coding	-3.0	-5.5
68	ZFYVE9	Coding	-2.9	-5.7
69	POU6F1	Coding	-2.4	-2.6
70	PRRT4	Coding	-2.3	-7.1
71	NAV1	Coding	-2.2	-8.1
72	SCMH1	Coding	-2.1	-1.8
73	SPTAN1	Coding	-2.1	-1.7
74	LPAR2	Coding	-1.9	-1.7
75	ZCCHC14	Coding	-1.8	-1.9
76	VGLL4	Coding	-1.4	-1.8

9 discordant

	Gene name	Transcript type	Linear FC PIF (19) vs PIR (175)	Linear FC NUP98+ (21) vs Other AML (137)
1	CLDN10	Coding	6.2	-13.0
2	CHRNB4	Coding	2.7	-9.1
3	NA	Coding	2.7	-2.2
4	AC244035.1	antisense	2.5	-5.5
5	LINC01006	processed transcript	2.2	-2.5
6	GCH1	Coding	-2.3	1.8
7	GASK1B	Coding	-3.4	3.0
8	STAB1	Coding	-3.5	3.8
9	DEFA1	Coding	-73.9	6.7

Table S1. **NUP98/PIF-associated DEGs**: 34 upregulated (on the left); 76 downregulated (in the middle); 9 discordant (on the right, excluded for next analysis).

TARGET						
**Gene symbol**	**Sensitivity (%)**	**Specificity (%)**	**PPV (%)**	**NPV (%)**	**AUC**	***p-value* AUC**
**RNU7-115P**	94.7	99.4	94.7	99.4	1.00	<0.0001
**AC092171.1**	94.7	93.1	60.0	99.4	0.97	<0.0001
**TRGV2**	84.2	85.7	39.0	98.0	0.87	<0.0001
**SPINK2**	78.9	86.3	38.5	97.4	0.89	<0.0001
**CCDC144CP**	89.5	82.9	44.7	98.6	0.93	<0.0001
**AP002812.2**	84.2	84.0	36.4	98.0	0.90	<0.0001
**TMA7**	100.0	100.0	100.0	100.0	1.00	<0.0001
**DEFA4**	78.9	70.9	22.7	96.9	0.79	<0.0001
**SPCS2**	94.7	100.0	100.0	99.4	0.99	<0.0001
**CDCP1**	52.6	89.7	35.7	94.6	0.76	0.0002
**ANP32E**	100.0	92.6	59.4	100.0	0.99	<0.0001
**LINC01881**	94.7	95.4	69.2	99.4	0.97	<0.0001
**AC026124.1**	73.7	78.9	27.5	96.5	0.76	0.0002
**CAPZA1**	89.5	94.9	65.4	98.8	0.95	<0.0001
**NT5C3AP1**	94.7	84.6	40.0	99.3	0.95	<0.0001
**PAFAH1B2**	84.2	86.9	41.0	98.1	0.91	<0.0001
**TRDMT1**	100.0	78.3	33.3	100.0	0.94	<0.0001
**LGALS12**	84.2	68.6	22.5	97.6	0.76	<0.0001
**CCR6**	47.4	89.7	33.3	94.0	0.64	0.0452
**NRG4**	89.5	68.6	23.6	98.7	0.78	<0.0001
**GNB5**	84.2	85.7	39.0	98.0	0.89	<0.0001
**FGFR1OP2**	84.2	90.3	48.5	98.1	0.92	<0.0001
**TMEM241**	94.7	78.3	32.1	99.3	0.92	<0.0001
**PCNT**	89.5	82.9	36.2	98.6	0.90	<0.0001
**NDUFA5**	78.9	95.4	65.2	97.7	0.93	<0.0001
**MAN1A2**	73.7	90.3	45.2	96.9	0.89	<0.0001
**GCNT1**	63.2	84.0	30.0	95.5	0.83	<0.0001
**NT5C3A**	89.5	88.6	45.9	98.7	0.93	<0.0001
**C9orf78**	100.0	86.3	44.2	100.0	0.97	<0.0001
**CPNE3**	94.7	68.6	24.7	99.2	0.84	<0.0001
**SRP54**	84.2	90.9	50.0	98.1	0.91	<0.0001
**CCDC25**	73.7	88.0	40.0	96.9	0.84	<0.0001
**CTR9**	94.7	61.1	20.9	99.1	0.82	<0.0001
**GOT1**	84.2	68.6	22.5	97.6	0.82	<0.0001

Table S2. A) **Sensitivity, Specificity, Positive Predictive Value (PPV) and Negative Predictive Values (NPV) for all 34 upregulated highly confident NUP98/PIF-associated DEGs**. All indices were calculated using best cut-off in Roc Curve for distinguishing PIF and PIR patients.

Gene symbol	Sensitivity (%)	Specificity (%)	PPV (%)	NPV (%)	AUC	*p-value* AUC
**CACNA2D2**	68.4	51.4	13.3	93.8	0.64	0.0505
**CELSR3**	84.2	73.1	25.4	97.7	0.86	<0.0001
**SCMH1**	89.5	34.3	12.9	96.8	0.68	0.0102
**USP2**	89.5	28.0	11.9	96.1	0.52	0.7291
**FLT4**	68.4	80.6	27.7	95.9	0.81	<0.0001
**TRIO**	89.5	47.4	15.6	97.6	0.71	0.0022
**HDAC7**	94.7	80.6	34.6	99.3	0.91	<0.0001
**LPAR2**	68.4	55.4	14.3	94.2	0.58	0.2325
**GRAMD1A**	100.0	88.6	48.7	100.0	0.96	<0.0001
**DFNB31**	78.9	64.6	19.5	96.6	0.74	0.0007
**PALM**	94.7	25.1	12.1	97.8	0.56	0.4286
**LRP5L**	5.3	62.9	1.5	85.9	0.62	0.0897
**ABCD1**	31.6	89.7	25.0	92.4	0.90	<0.0001
**TSC2**	94.7	88.0	46.2	99.4	0.94	<0.0001
**CAPN15**	100.0	74.9	30.2	100.0	0.92	<0.0001
**CLIP3**	94.7	42.9	15.3	98.7	0.68	0.0095
**DNM1**	89.5	44.0	14.8	97.5	0.72	0.0021
**GLIS3**	47.4	88.0	30.0	93.9	0.68	0.0097
**SHB**	73.7	70.3	21.2	96.1	0.75	0.0003
**AADAT**	68.4	86.3	35.1	96.2	0.81	<0.0001
**ARHGEF17**	57.9	88.0	34.4	95.1	0.79	<0.0001
**PODXL2**	94.7	66.3	23.4	99.1	0.87	<0.0001
**TANC1**	47.4	42.9	8.3	88.2	0.51	0.8922
**XPNPEP2**	68.4	89.7	41.9	96.3	0.81	<0.0001
**PKN1**	73.7	80.6	29.2	96.6	0.81	<0.0001
**KDM4B**	89.5	57.1	18.5	98.0	0.72	0.0016
**MMP28**	10.5	69.1	3.6	87.7	0.51	0.8549
**GPR12**	68.4	71.4	20.6	95.4	0.70	0.0034
**NAV1**	89.5	29.1	12.1	96.2	0.53	0.6499
**DAGLA**	94.7	36.6	14.0	98.5	0.68	0.0114
**ALPK3**	73.7	94.3	58.3	97.1	0.89	<0.0001
**PAQR5**	52.6	87.4	31.3	94.4	0.71	0.0028
**ZCCHC14**	84.2	36.0	12.5	95.5	0.55	0.4633
**HSPG2**	94.7	80.0	34.0	99.3	0.92	<0.0001
**VGLL4**	10.5	69.1	3.6	87.7	0.55	0.4425
**TNFRSF21**	94.7	43.4	15.4	98.7	0.67	0.0133
**RNF217**	73.7	64.0	18.2	95.7	0.69	0.0069
**STK32B**	78.9	57.7	16.9	96.2	0.67	0.0146

Gene symbol	Sensitivity (%)	Specificity (%)	PPV (%)	NPV (%)	AUC	*p-value* AUC
**KCTD15**	57.9	85.7	30.6	94.9	0.76	0.0002
**ROBO4**	57.9	87.4	33.3	95.0	0.67	0.0148
**ENPP3**	89.5	22.9	11.2	95.2	0.72	0.0013
**VSIG4**	63.2	56.0	13.5	93.3	0.52	0.7814
**ZFYVE9**	100.0	87.4	46.3	100.0	0.59	0.1761
**SEPN1**	78.9	58.9	17.2	96.3	0.97	<0.0001
**KIF26B**	73.7	37.1	11.3	92.9	0.71	0.0022
**HPGDS**	89.5	50.9	16.5	97.8	0.53	0.6546
**FBLN2**	47.4	81.7	22.0	93.5	0.73	0.0008
**ZNF704**	94.7	58.3	19.8	99.0	0.65	0.0381
**ANXA8**	68.4	74.3	22.4	95.6	0.76	0.0002
**ARHGEF40**	57.9	92.0	44.0	95.3	0.72	0.0015
**DCHS1**	94.7	70.3	25.7	99.2	0.77	0.0001
**CENPV**	42.1	90.3	32.0	93.5	0.88	<0.0001
**LOXHD1**	68.4	89.7	41.9	96.3	0.66	0.0266
**RCOR2**	100.0	74.3	29.7	100.0	0.83	<0.0001
**MAP4K2**	42.1	78.3	17.4	92.6	0.90	<0.0001
**DCLK2**	68.4	65.7	17.8	95.0	0.57	0.3277
**TRH**	47.4	69.1	14.3	92.4	0.68	0.0111
**EVC2**	100.0	95.4	70.4	100.0	0.57	0.3214
**PACS1**	78.9	60.6	17.9	96.4	0.99	<0.0001
**ZNF467**	68.4	82.9	30.2	96.0	0.71	0.0025
**GPR88**	89.5	68.0	23.3	98.3	0.79	<0.0001
**TRAK1**	89.5	38.9	13.7	97.1	0.80	<0.0001
**POU6F1**	26.3	82.9	14.3	91.2	0.64	0.0476
**LPAR5**	63.2	71.4	19.4	94.7	0.85	<0.0001
**SLC24A3**	89.5	62.3	20.5	98.2	0.71	0.0027
**ATP2A1**	84.2	68.0	22.2	97.5	0.73	0.0008
**NTNG2**	89.5	53.1	17.2	97.9	0.80	<0.0001
**SPTAN1**	89.5	37.1	13.4	97.0	0.62	0.0938
**RD3**	78.9	44.0	13.3	95.1	0.62	0.0753
**VIT**	31.6	90.9	27.3	92.4	0.61	0.1189
**SAP25**	78.9	27.4	10.6	92.3	0.89	<0.0001
**AC104809.2**	73.7	49.7	13.7	94.6	0.52	0.7259
**PRRT4**	84.2	50.9	15.7	96.7	0.62	0.0865
**RP11-403I13.9**	68.4	64.0	17.1	94.9	0.66	0.0206
**RP11-510H23.1**	84.2	62.3	19.5	97.3	0.61	0.1105
**RP11-473M20.7**	78.9	53.1	15.5	95.9	0.73	0.0008

Table S2. B) **Sensitivity, Specificity, Positive Predictive Value (PPV) and Negative Predictive Values (NPV) for all 76 downregulated highly confident NUP98/PIF-associated DEGs**. All indices were calculated using best cut-off in Roc Curve for distinguishing PIF and PIR patients.

Gene name	TARGET program Linear FoldChange NUP98+ vs NUP98-	Adj. *p-value*	TARGET program Linear FoldChange PIF vs PIR	Adj. *p-value*	Noort Study Linear FoldChange NUP98-NSD1+ vs NUP98-NSD1-	Adj. *p-value*
**Upregulated**						
**NRG4**	3.6	0.0006800	2.4	0.0073354	15.9	2.30E-16
**DEFA4**	5.8	0.0006774	4.3	0.0016807	3.7	0.0003144
**SPINK2**	4.7	0.0002229	8.3	2.16E-11	19.6	8.01E-14
**CPNE3**	2.1	0.0006891	1.9	0.0032273	2.1	0.0000009
**LGALS12**	2.1	0.0229131	2.6	0.0001047	3.1	1.38E-08
**CDCP1**	2.4	0.0268617	4.0	4.13E-07	8.1	1.82E-15
**RP11-305O6.3**	2.5	0.0042777	3.6	6.47E-07	2.9	0.0000095
**GCNT1**	2.0	0.0044462	2.1	0.0002831	2.7	1.74E-12
**Downregulated**						
**XPNPEP2**	-6.8	0.0025736	-20.5	7.21E-07	-3.5	1.18E-09
**RCOR2**	-10.4	7.79E-06	-17.6	3.62E-08	-5.9	6.36E-12
**CELSR3**	-2.2	0.0260854	-10.1	1.74E-11	-2.1	2.69E-07
**FLT4**	-55.3	9.77E-09	-20.1	1.40E-05	-4.4	0.0000051
**DFNB31**	-7.4	7.30E-11	-4.8	1.06E-06	-2.8	3.81E-09
**FBLN2**	-2.9	0.0345421	-5.4	0.0007283	-4.1	1.41E-07
**VIT**	-41.4	0.0027521	-17.7	0.0090988	-2.6	0.0001133
**GPR12**	-66.5	2.31E-06	-10.2	0.0044818	-6.1	1.39E-07
**EVC2**	-17.9	0.0010810	-7.9	0.0126903	-4.0	0.0000072
**PRRT4**	-7.1	1.75E-06	-2.3	0.0243112	-3.4	0.0000033
**POU6F1**	-2.6	1.98E-06	-2.4	3.83E-05	-2.6	1.72E-16
**GLIS3**	-20.5	0.0001184	-11.1	0.0005791	-4.0	0.0000048
**LOXHD1**	-11.8	4.05E-05	-3.1	0.0470452	-2.7	0.0057301
**DCHS1**	-7.4	3.05E-07	-5.8	1.70E-05	-4.3	6.37E-12
**DCLK2**	-5.1	0.0132501	-3.8	0.0219993	-3.6	0.0000083
**ALPK3**	-5.2	0.0016214	-19.6	1.52E-08	-3.4	1.64E-08
**RP11-403I13.9**	-4.6	0.0467955	-5.5	0.0117711	-3.0	0.0000176
**CACNA2D2**	-9.7	0.0001959	-4.8	0.0031621	-5.1	4.90E-10
**RP11-510H23.1**	-6.3	0.0053864	-4.0	0.0278720	-3.6	0.0000011
**LPAR5**	-5.8	1.23E-07	-8.8	2.59E-09	-2.3	5.94E-08
**ZFYVE9**	-5.7	0.0001245	-2.9	0.0154511	-4.7	4.19E-10
**USP2**	-5.5	0.0032263	-3.0	0.0401788	-7.1	1.02E-14
**KCTD15**	-2.6	0.0016137	-4.7	1.78E-07	-3.3	9.71E-10
**TANC1**	-10.9	4.79E-05	-3.2	0.0214368	-2.4	0.0011365
**NAV1**	-8.1	9.53E-08	-2.2	0.0496939	-2.6	0.0000136
**AADAT**	-6.5	0.0013006	-9.5	0.0003850	-10.9	4.53E-13
**RNF217**	-3.1	0.0399202	-4.0	0.0043638	-6.1	3.60E-08
**ZNF704**	-5.0	0.0054430	-3.8	0.0065976	-28.1	4.03E-29
**SLC24A3**	-4.1	0.0234298	-7.0	0.0014564	-3.1	0.0005889
**TRH**	-8.1	0.0066287	-5.9	0.0090749	-2.7	0.0402381
**HPGD**	-7.1	0.0011962	-3.7	0.0108997	-10.6	7.78E-18

Table S3. **Thirty-nine differentially expressed genes in three different groups: NUP98+ vs NUP98- and PIF vs PIR from TARGET program, then NUP98-NSD1+ vs NUP98-NSD1- from Noort study.** We show 8 upregulated and 31 downregulated transcripts, obtained by crossing our list of 110 NUP98/PIF-associated DEGs and 3026 NUP98-NSD1 differentially expressed genes reported by Noort et al.

	AIEOP LAM 2002/01	TARGET PROGRAM		LOCAL COHORT	
	Total AML patients	PIF	PIR	PIF	PIR
**Patients, *n (%)***	482	19	175	4	8
**Gender (male/female)**	262 (52%)/ 220	12 (63%)/ 7	91(52%)/ 84	0/4	4/4
**Age at diagnosis**					
**< 1 year**	63 (13%)	0	8 (4%)	0	0
**1-2 years**	52 (10%)	3 (16%)	17 (10%)	0	0
**2-10 years**	181 (38%)	8 (42%)	63 (36%)	2 (50%)	3 (37.5%)
**> 10 years**	186 (39%)	8 (42%)	87 (50%)	2 (50%)	5 (62,5%)
**WBC at diagnosis (×10⁹/l)**					
**<10**	171 (35%)	2 (10%)	19 (11%)	2 (50%)	5 (62,5%)
**10-99**	236 (49%)	10 (53%)	116 (66%)	2 (50%)	3 (37,5%)
**>100**	75 (16%)	7 (37%)	40 (23%)	0	0
**FAB Subtype**					
**M0**	34 (7%)	3 (16%)	1 (0,6 %)	0	0
**M1**	88 (18%)	1 (5%)	25 (14%)	3 (75%)	2 (25%)
**M2**	91 (19%)	4 (21%)	42 (24%)	1 (25%)	2 (25%)
**M4**	83 (17%)	2 (11%)	44 (25%)	0	2 (25%)
**M5**	117 (24%)	3 (16%)	33 (19%)	0	1 (12,5%)
**M6**	5 (1%)	0	3 (2%)	0	0
**M7**	44 (9%)	1 (5%)	6 (3,4%)	0	1 (12,5%)
**Unclassifiable/not known**	20 (4%)	5 (26%)	21 (12%)	0	0
**Patients with available cytogenetic data**	418				
**Normal**	166 (39%)	7 (37%)	46 (26%)		
t(8;21), t(16;16) or inv(16)	99 (24%)	1 (5%)	55 (31%)	0	0
**t(9;11) or MLL-t**	65 (15%)	2 (11%)	31 (18%)	0	0
**NUP98-rearrangements**	25 (5%)	4 (21%)	12 (7%)	1 (25%)	4 (50%)
**Other**	61 (15%)	9 (47%)	34 (19%)	1 (25%)	6 (75%)
**Complex karyotype***	27 (6%)	1 (5%)	23 (13%)	1 (25%)	2 (25%)
**Unknown**	0	0	9 (5%)	1 (25%)	0
**Patients tested for molecular aberrations**	384				
***FLT3*-ITD**	42 (11%)	3 (16%)	21 (12%)	0	5 (62,5%)
***FLT3* activating loop mutations**	10 (3%)	2 (11%)	14 (8%)	0	0
**Other molecular aberration**	244				
***NPM1* mutations**	14 (6%)	0	11 (6%)	0	0
***CEBPA* mutations**	18 (7%)	0	17 (10%)	0	0
WT1	NA	4 (21%)	9 (5%)	0	0
****Complex Karyotype, ≥ 3 abnormalities (numerical or structural)***					

Table S4. **Characteristics of patients with AML included in our study**. PIF: Primary Induction Failure; PIR: Primary Induction Response; Number of cases (% of cases per category); N.A: not available; * Complex karyotype: ≥ than 3 chromosomal abnormalities numerical or structural).

	TARGET PROGRAM	
	NUP98r+	NUP98r+
**Patients, *n (%)***	21	137
**Gender (male/female)**	15/ 6	70/ 67
**Age at diagnosis**		
**< 1 year**	0	11
**1-2 years**	3	13
**2-10 years**	9	46
**> 10 years**	9	67
**WBC at diagnosis (×10^9/l)**		
**<10**	4	14
**10-99**	11	89
**>100**	6	34
**FAB Subtype**		
**M0**	0	3
**M1**	2	11
**M2**	7	35
**M4**	4	37
**M5**	4	34
**M6**	2	0
**M7**	1	4
**Unclassifiable/not known**	1	13
**Overall Survival (days)**	1214	Undefined
**Patients with available cytogenetic data**		
**Normal**	12	7
t(8;21), t(16;16) or inv(16)	0	68
**t(9;11) or MLL-t**	0	39
**NUP98-rearrangements**	21	0
**Other**	7	20
**Complex karyotype***	3	11
**Unknown**	1	3
**Patients tested for molecular aberrations**		
***FLT3*-ITD**	9	7
***FLT3* activating loop mutations**	0	10
**Other molecular aberration**		
***NPM1* mutations**	0	0
***CEBPA* mutations**	1	1
WT1	6	6

Table S5. **Clinical and pathological features and molecular alterations of selected TARGET samples, analyzed by transcriptome analysis and subdivided in NUP98r positive and NUP98r negative patients**

Transcript (Accession number)	Forward Primer	Reverse Primer	Annealing Temperature	Fragment size
SPINK2 (NM_001271718.2)	2F-TCCCTCAATTTGGTCTGTTTTC	3R-CCTTCCCTGATTTTCATGCAC	60°C	151 bp
TMA7 (NM_183250.2)	2F-AGAAGCAGGCCAAGGAGATG	4R GCCAGATTTCTTAATTCCACCTG	59°C	140 bp
SPCS2 (NM_014752.3)	2F TGACAAGTGGGATGGATCAGC	3R TACAGATGGTGAGGCGACCA	58°C	122 bp
CDCP1 (NM_022842.5)	4F-TCAACCTCTCTCTGCAAGGC	5R-CGCTCATTACTCAAGTCAACC	58°C	133 bp
CAPZA1 (NM_006135.3)	7F ACCATCACACCACCTACAGC	9-10R TCACTAATTGCTGTCTGATACTCAT	60°C	197 bp
FGFR1OP2 (NM_015633.3)	3F-ACGGTCCACGTTAGTTATGG	4R-AAGTTCCAAGGCCGACTGAT	56°C	115 bp
MAN1A2 (NM_006699.5)	12F ATTGCCGAGTTAATGGTGGGT	13R TCGAACAGCAGGATTACCTGA	57°C	233 bp
NT5C3A (NM_001002010.5)	7F-AACATAGCATCCCCGTGTTCA	7/8R-AATCCTTTGAGCACCCCAGTT	57°C	145 bp
SRP54 (NM_003136.4)	9F CATGCAAAAGGAGGTGGTGC	11R TCAGTCCTTCAATGTCGCCC	57°C	154 bp
MALAT1 (NR_002819.4)	3endF-AAAGCAAGGTCTCCCCACAAG	3endF-GGTCTGTGCTAGATCAAAAGGCA	61°C	73 bp

Table S6. Primers details used for qRT-PCR
